# Supplementary material for: Experiences of redeployed healthcare workers in the fight against COVID-19 in China: A qualitative study
Source: PLoS One. 2022 Aug 25;17(8):e0273429. doi: 10.1371/journal.pone.0273429 (PMC9409527; doi:10.1371/journal.pone.0273429)
Supplement: S1 File — Understanding your needs and experiences of caring COVID-19 patients. (DOCX) [file pone.0273429.s001.docx]

S1 File

**Topic Guide for Staff Interview Participants**

**Understanding your needs and experiences of caring COVID-19 patients**

**Introduction**

Thank you so much for agreeing to be interviewed today. My name is _________ and I am a member of the team working on this study. The purpose of today’s interview is to understand your needs and experiences of caring for COVID-19 patients. This will provide important insight into meaningful targets for improvements in quality of care during the current pandemic and recommend preparedness plans for future pandemics.

The interview should take about 30-60 minutes. The information you share in this interview will be anonymized, so please feel free to share as much as you would like to. We may cover some difficult/challenging topics during the interview. You can stop the interview at any point or skip any questions should you wish to. Also, we can pause for breaks if you need to, and can restart recording when you are ready.

Could I please just confirm that you have signed consent form and read the information sheet? And are you happy for the interview to be audio recorded today? And is it ok for me to make a few notes during the interview?

Do you have any questions before we start?

****Start recording****

1. Could you please tell me about yourself?
2. We are interested in your real experiences of providing direct care for COVID-19 patients. Could you please tell me about it?

Prompts:

How has the experiences of fighting against COVID-19 affected you personally and professionally?

What support have you received to help you with providing direct care for COVID-19 patients?

If you were deployed to Wuhan, what are your unique experiences of working with new colleagues in a new environment in the epicentre of the pandemic?

1. What are your priorities when being asked to care for COVID-19 patients?

Prompts:

Can you tell us about any training and preparation you may have had about the care and treatment of COVID-19?

How do you balance between professional obligation to care of patients and personal risks/ risks of your families?

What precautions do you institute to optimise your families’ safety (eg. sending their families away or eliminating social interactions)?

1. What are your main needs and concerns while caring for COVID-19 patients during the pandemic?
2. It must be very challenging to care for patients with COVID-19. Could you please tell me about any distress being experienced?

Prompts:

What are the issues and challenges you have faced? How have you coped with these difficulties and challenges?

Is there anything especially hard to cope with? What strategies have you used to help you to cope with these challenges during the current pandemic?

1. We are particularly interested in your experiences of and views towards caring for critically ill COVID-19 patients including those approaching end of life. Could you please tell me more about it?

Prompts:

What would you say were the most important things that the patients value at end of life at this challenging moment?

What were their main symptoms? How did you manage these symptoms?

What were their multidimensional needs (physical, psychological, social, spiritual, information) and how were these needs met?

What were the main problems and main concerns of those patients?

What kind of support have you delivered to these patient’s family?

How did you communicate with patients and their families about their disease, treatment, and bad news?

What challenges have you faced when caring for those patients at end of life? How have you done to cope with these challenges?

How have you and your colleagues dealt with difficult conversations and decision making towards life sustaining treatment and the use and withdrawal of life sustaining equipment?

1. I would like to know about how health services have responded to the current COVID-19 pandemic. Could you please share with me about your experience of it?

Prompts:

How has normal delivery of care been changed to respond the current pandemic?

What changes have been made to respond the current COVID-19 pandemic?

What further changes would you like to make to the healthcare services or system to ensure a more effective and efficient quality of care responsive to the current pandemic?

Are there any changes of physician or nurse-patient relationship? If so, how?

How did you communicate with patients and their families about their disease and treatment?

How do you think patients made choices about his/her health?

Their decision-making preferences and involvement in decision making process?

When the patients’ health conditions worsened and they are approaching end of life, would you recommend them to receive palliative care?

What are your views towards palliative care in situations when the lives of patients with severe COVID-19 could not be saved?

How can palliative and end of life care be promoted and improved in China?

1. Could you please tell me about any areas for improvement to make more patient-centred care which will better address individual needs in response to pandemics?

Prompts:

How do you see pandemic changing in the near future/further ahead?

What are your main concerns and thoughts about the future pandemics?

What should we do to better prepare ourselves for future pandemics e.g., training?

What are your recommendations for policy makers, hospital managers, community-based health services, healthcare providers, and the general public to improve preparedness for future pandemics?

1. Is there anything you would like to add about your experiences of care or anything we have missed out/not spoken about? You can always contact us after if there is anything you would like to add.

I’d like to thank you for taking the time to be interviewed today, we really appreciate it and your views will be a great help to us.

****End recording****
