# Supplementary material for: Experiences of redeployed healthcare workers in the fight against COVID-19 in China: A qualitative study
Source: PLoS One. 2022 Aug 25;17(8):e0273429. doi: 10.1371/journal.pone.0273429 (PMC9409527; doi:10.1371/journal.pone.0273429)
Supplement: Attachment — Submitted filename: Response to reviewers PLOS ONE R1 08.04.22.docx [file pone.0273429.s003.docx]

**Experiences of redeployed healthcare providers in the fight against COVID-19 in China:**

**a qualitative study**

Response to editors/reviewers

| **Reviewers’ comments** | **Authors’ response and changes made** |
| --- | --- |
| Editor(s)' Comments to Author: | |
| The research is of great significance in the fight against COVID-19 in China. But paper structure including introduction, discussion, and conclusion sections, is confused and should be improved. In the method section, more details of sample, data, variables, and data statistics are needed in the revised version. | Thank you for recognising the significance of our work in the fight against COVID-19 in China. We have revised paper structure and provided more details in the methods section as suggested. Please see our specific revisions made below. |
| Theoretical framework is weak and the previous relevant COVID-19 references should be cited to provide better implications of management of healthcare. | Thank you very much for your comments. We understand that the theoretical framework is one of the most important aspects in qualitative research processes. When we designed this study, we considered different theoretical frameworks which could be used. However, we decided not to use a theoretical framework as we analysed qualitative data inductively, which means the analysis should be conducted without any theoretical framework, preconception, and advance knowledge.  We have read and used all the relevant COVID-19 references to strengthen the implications in healthcare management. |
| Referee 1 | |
| As the author mentioned that the participants attended COVID-19 isolation wards in Wuhan from 26th January to 26th March 2020 (60 days), but the Face-to-face, semi-structured in-depth interviews were conducted 1st September 2020 and 31st May 2021. There had a long time interval, which may impact the feeling of the participants. | Thank you very much for your helpful comments. We agree that the time interval between redeployment and interviews which may impact the recall and feelings of the participants. However, after having carefully considered this and consulted with local research/ clinical team members in the study design phase, 6 to 14 months’ period was considered as the most appropriate time interval because:   - The focus of our study was to not only capture the feeling of the participants, but to also explore the long-term impacts of their redeployment experiences on their work and life. An appropriate time interval should be long enough to allow participants to have the opportunity to introspect and reflect the experience of deployment and caring for COVID-19 patients at the epicentre but should not be so long to avoid recall bias. - 6 to 14 months’ time interval could help to avoid or minimise unnecessary distress and worries which might be potentially induced by interviewing healthcare providers immediately after they returned from Wuhan. - The time interval in this study also allowed researchers to conduct face to face interviews which promoted the study participation and generated richer data around this topic than online interviews. |
| The introduction should be rewritten, which should describe the purpose and significance of this study. | Thank you so much for pointing this out. We have rewritten some parts of introduction, and added more background information in this section to further justify the significance of this study as below:  **COVID-19 is characterised by high transmission rates, at times leading to large outbreaks, is triggering public health responses[1, 2]. Hundreds of millions of individuals infected worldwide, leading to the death of millions and a substantial indirect influence on other diseases and posing major public health and governance challenges in terms of economic, medical and health infrastructure[3-7]. People with chronic underlying diseases are vulnerable with the virus and would experience worse outcomes. Severe disease can lead to heart, and respiratory failure, acute respiratory syndrome, or even death as well as serious psychological outcomes[8-10].** (Line 70-79)  **The rapid escalation in the number of infections have resulted in insufficient healthcare resources[11]****. The potentially overwhelming burden of illnesses stresses health system capacity and brings adverse effects to healthcare providers, including the risk of infection[12-14]. There will probably be a high burden of COVID-19 in countries where there are fragile healthcare systems, lack of access to clean water and disinfectants,** **poor outbreak preparedness, severe shortages in personal protective equipment (PPE) and medical technology, challenges in enforcing physical distancing regulations, and reliance on informal employment[15]. In such countries,** **it is expected that patients with severe COVID-19 who are unable to receive acute care in hospitals will suffer and die, being cared for by family members without PPE and access to relevant information, training, or palliative care resources[16].** (Line 80-90)  …  **Healthcare providers at the front line of the COVID-19 outbreak are exposed to hazards that put them at risk of infection. As of 14^th^ February, 1716 healthcare providers were confirmed in China [31]. Overall, 14.8% of confirmed cases among healthcare providers were classified as severe or critical and 5 deaths were observed [31]. In addition, healthcare providers caring for COVID-19 inpatients were under extraordinary stress related to high risk of stigmatization, understaffing, and uncertainty [32]. Quantitative studies have shown that frontline healthcare providers treating patients with COVID-19 have greater risks of mental health problems, such as anxiety, depression, insomnia, and stress [33]. Transmission from healthcare providers to their family members is widely reported [34]****.** (Line 124-132)  We have revised to clearly described the purpose of this study as below:  **We aimed to (1) explore the experiences and needs of redeployed frontline HCWs caring for COVID-19 patients in Wuhan, China; and (2) understand the long-term impacts of this redeployment experiences on their work and life. The specific objectives were:**  **Objective 1****. To explore distress being experienced, the issues and challenges being faced, and their ways of coping with these from healthcare providers’ perspectives.**  **Objective 2. To explore experiences and views towards caring for COVID-19 patients at end of life (e.g., how to address the patients’ symptoms, multidimensional needs, main problems and concerns).**  **Objective 3. To explore current delivery of care and health system changes in response to COVID-19 and identify areas for improvement to make more patient-centred care and make recommendations for policy makers and healthcare providers to improve preparedness for future pandemics.** (Line 140-151) |
| The results of the main found should be expressed using tables, the format of present was not suitable for a paper. | Thank you for this suggestion. We have presented the themes and subthemes identified from interviews in Table 2. We have indeed used tables to present some representative quotes when we have word limit challenges, but would prefer to keep the data in the text in this instance as it can create an easy flow for the reader to see the data within the text. |
| The discussion should focus on the main new discovery of this manuscript and compared with other previous studies. | In our discussion, we have focused on the following main new discoveries which were directly drawn from the interview data:   - Psychological impact of the participants’ redeployment experience - The challenges they faced caring for COVID-19 patients in the context of resource strain in Wuhan and strategies to overcome those challenges - Support needed - Their professionalism and patriotism   We have compared our findings with other previous studies. For example,  **Support of redeployed HCWs has attracted extensive attention worldwide[40-43]. NHS England and NHS Improvement issued national guidance on key considerations for the safe redeployment of staff and deployment of those joining the NHS temporarily to support the existing workforce [44]. United States Centres for Disease Control and Prevention provided guidance for redeployed teams on capacity development at the national and sub-national levels during an active COVID-19 response [45].** (Line 560-566)  **…**  **These results were consistent with previous studies from Chinese cultural contexts [49-52]. Cultural factors take an important role in the delivery of palliative care [53]. To a large extent, HCWs’ beliefs and attitudes towards health and disease are influenced or determined by their traditional culture. Talking about death and dying and giving up positive treatment is very challenging for HCWs [54, 55]. Discussions about death are often avoided as they are seen hastening the pace of patients’ dying process and incurring bad luck [56, 57].** (Line 582-588) |
| Referee 2 | |
| The topics of this paper are interesting, but the structure and content must be revised, and results have to be better explained by authors before to be reconsidered for publication. | Thank you for recognising our work is interesting. We have revised paper structure and content to convey our messages more clearly as suggested. |
| Title has to be shorter and fit better the topics under study. | We have shortened our title to better fit the study as below:  **Experiences of redeployed healthcare providers in the fight against COVID-19 in China: a qualitative study** |
| Abstract has to be shorter focusing on results, and health and social implications of this study for improving healthcare management. | We have revised the abstract as suggested (Line 34-35; Line 59-66). |
| Introduction has to better clarify the research questions of this study and provide more theoretical background. | We have revised our aim of this study to make it clearer. As we responded earlier, we understand that the theoretical framework is one of the most important aspects in qualitative research processes. When we designed this study, we considered different theoretical frameworks which could be used. However, we decided not to use a theoretical framework as we analysed qualitative data inductively, which means the analysis should be conducted without any theoretical framework, preconception, and advance knowledge. |
| Authors have to better describe the different sources of transmission dynamics of COVID-19 and risk factors in society, as well as effects of the policy of lockdown on organizations and society in China (See suggested readings that must be all read and used in the text). | This is a very helpful comment and thank you. We have read and used all the 23 suggested papers. We have added better description about the different sources of transmission dynamics of COVID-19 and risk factors in society (Line 70-90):  **COVID-19 is characterised by high transmission rates, at times leading to large outbreaks, is triggering public health responses[1, 2]. Hundreds of millions of individuals infected worldwide, leading to the death of millions and a substantial indirect influence on other diseases and posing major public health and governance challenges in terms of economic, medical and health infrastructure[3-7]. People with chronic underlying diseases are vulnerable with the virus and would experience worse outcomes. Severe disease can lead to heart, and respiratory failure, acute respiratory syndrome, or even death as well as serious psychological outcomes[8-10].**  **The rapid escalation in the number of infections have resulted in insufficient healthcare resources [11]. The potentially overwhelming burden of illnesses stresses health system capacity and brings adverse effects to healthcare providers, including the risk of infection [12-14]. There will probably be a high burden of COVID-19 in countries where there are fragile healthcare systems, lack of access to clean water and disinfectants, poor outbreak preparedness, severe shortages in personal protective equipment (PPE) and medical technology, challenges in enforcing physical distancing regulations, and reliance on informal employment [15]. In such countries, it is expected that patients with severe COVID-19 who are unable to receive acute care in hospitals will suffer and die, being cared for by family members without PPE and access to relevant information, training, or palliative care resources [16].**  Regarding the effects of the policy of lockdown on organizations and society in China, we have included some relevant contents in the introduction section of our original paper so there is no further change made (Line 92-106). For example,  At the early stage, in the face of a previously unknown virus, China rolled out the most ambitious, agile and aggressive disease containment effort in history [11, 12]. In Wuhan, Hubei province of China, the initial epicentre, general wards were quicky modified into isolation wards, and healthcare workers (HCWs) stepped up to provide care for patients with COVID-19 as ‘fever clinics’ and respiratory and infection disease units were overwhelmed by an increasing number of suspected and confirmed cases.  China launched the national emergency response and initiated a range of measures to mitigate the transmission of COVID-19 and reduce the impact of the outbreak on healthcare systems, including redeployment of healthcare resources to the most vulnerable populations and communities [13]. During the COVID-19 crisis, staff redeployment has been extensive and varied. Central government has continued to send over 42,000 HCWs (including over 28,600 nurses) and tons of vital personal protective equipment (PPE) supplies across China to Hubei province and Wuhan city [14]. Staff with different specialities have been redeployed into high-risk areas from their relatively safe ‘home’ wards [15]. |
| Methods of this study is not clear. The section of Materials and methods must be re-structured with following three sections: • Sample and data • Measures of variables • Data analysis procedure. Study design can be improved. Current sections can be reorganized in the just mentioned sections. | We have included the contents in relation to sample (participants), data collection, and data analysis with clear sub-headings in our methods section. Measures of variables is not applicable in our work because this is not a quantitative study. We can confirm that we reported this paper in accordance with the consolidated criteria for reporting qualitative research (COREQ) guidelines. More information about sample and data collection has been added (Line 176-186; Line 190-195). |
| Results are confused. Although the study is qualitative, I suggest synthetizing and systematize results in tables showing main findings according to the categorization of working place/department (Emergency, ICU, Respiratory and infectious disease), marital status and gender to detect similarity and/or differences of effects on working condition and mental health to provide better implications of management of healthcare. | We have reformatted the demographic table and synthesised the data more clearly (see Table 1). This revised demographic table clearly shows working place/department of the participants (Emergency, ICU, respiratory and infectious disease), and their marital status and gender. This qualitative study was not designed to detect the similarity and/or differences of effects on working condition and mental health across groups. So this is beyond the scope of our study. |
| As scholars this is the research activity to do with this qualitative information to extend the knowledge in these topics. | We agree that our qualitative research extended the knowledge in this topic. |
| Results with these table can be synthetized better instead of presenting all sentences. | We have presented the themes and subthemes identified from interviews in Table 2. We presented our findings using some direct quotations from research participants which is recommended by the consolidated criteria for reporting qualitative research (COREQ) guidelines. As we responded to an earlier point, we have indeed used tables to present some representative quotes when we have word limit challenges, but would prefer to keep the data in the text in this instance as it can create an easy flow for the reader to see the data within the text. |
| Discussion can remove sub-headings that create fragmentation and confusion. | We have removed the sub-heading in the discussion section as suggested. |
| Discussion to be fruitful and useful to cope with future pandemics have to support implications of crisis management of human resource, using theories of self-determination, intrinsic and extrinsic incentives to reduce negative effects in organizations in problematic situation (suggested papers can support a similar and fruitful discussion) to increase the impact of this paper and utility for readers of the journal, such as managers and policymakers. Discussion should also consider similar problems in other countries (see suggested papers). | This is a very helpful comment and thank you. We have strengthened the implication of crisis management of human resource for wider audience by using theories of self-determination. Relevant references from different countries have been cited. Please find details below:  **The reality of a pandemic response may reduce HCWs’ willingness to work due to varieties of reasons including inadequate preparedness of facility and workplace safety[70]. Voluntary absenteeism may be caused by fear of infection, duties to one’s family and loved ones, lack of resources and trust in the management of facilities and government[71]. It is important to motivate HCWs to work continuedly during pandemic and to promote crisis management of human resource and relevant training [72]. Theories of self-determination[73] and intrinsic and extrinsic incentives[74] can be used to suggest management implications directed to support motivation and performance of HCWs in the crisis[75-80].**  **Maintaining an adequate healthcare workforce in this crisis requires not only an adequate number of physicians, nurses, advanced practice clinicians, pharmacists, respiratory therapists, and other clinicians, but also maximizing the ability of each clinician to care for a high volume of patients [91]. Given that surges in critically ill patients could last weeks to months, it is also essential that healthcare providers are able to perform to their full potential over an extended time. In order to effectively support frontline healthcare providers and address the challenges they are facing during the pandemic, it is critical to understand their experiences and needs in the fight against COVID-19 in China [92].** (Line 636-653) |
| Conclusion is poor and must be rewritten and extended. It has not to be a summary, but authors must focus on manifold limitations of this study and provide suggestions of health, crisis management and social policy. As well as strategies of preparedness and prevention of future pandemic diseases (see suggested docs). | The limitations of this study have been included in our discussion section of the original paper. The key implications have been discussed in the discussion section too. Therefore, we would prefer a concise conclusion. As suggested, we have rewritten the conclusion section to highlight important implications as below:  **This qualitative study comprehensively demonstrated how redeployed HCWs in the initial epicentre of COVID-19 in China were impacted by being on the front line of a pandemic. HCWs shared unique experiences and a range of challenges in the context of resource strain which had significant implications in policy and future practice. The themes constructed in this study can be used by managers and policy makers to mitigate negative psychological outcomes of HCWs and promote emergency and logistic preparedness and response. Continuously evolving pandemic circumstances and a sense of uncertainty expressed by our participants highlight the importance of an organized national pandemic response plan for subsequent waves of COVID-19 and future pandemics.** (Line 671-680) |
| Overall, then, the paper is interesting, but structure is confused. Theoretical framework is weak, and some results create confusion… structure of the paper must be improved; study design, discussion and presentation of results have to be clarified using suggested comments. If the paper is improved, by using all comments, maybe it can be considered. Suggested readings of relevant papers that must be read, and all inserted in the text and references. | Thank you again for all your constructive comments. We have revised the paper structure and contents of some sections to enhance the clarity of our paper.  All your comments have been carefully considered by the project team and addressed accordingly.  Thank you so much for suggesting relevant papers. We have read and used all the 23 suggested papers. |
| Referee 3 | |
| The aim of this study was to explore the experiences and needs of frontline healthcare workers who were redeployed to care for COVID-19 patients in Wuhan, China. This is a well-written and well-presented article on an important topic. | Thank you very much for recognising the significance and impact of our work. We appreciate your positive comments on our writing quality and presentation of this paper. |
| This research is qualitative and shows no statistical data. | Thank you so much for pointing this out. Our study is qualitative, which does not include statistical data. We employed interviews to get rich and insightful data to enable us to make important recommendation and conclusions for future practice and policy marking. |
| I would expect to see a better conclusion at the end of the manuscript. | To respond the same comment from another reviewer, we have strengthened the conclusion section (Line 671-680). |
| Journal Requirements | |
| When submitting your revision, we need you to address these additional requirements.  1. Please ensure that your manuscript meets PLOS ONE's style requirements, including those for file naming. The PLOS ONE style templates can be found at  <https://journals.plos.org/plosone/s/file?id=wjVg/PLOSOne_formatting_sample_main_body.pdf> and <https://journals.plos.org/plosone/s/file?id=ba62/PLOSOne_formatting_sample_title_authors_affiliations.pdf> | I can confirm that our manuscript meets PLOS ONE’s style requirement. |
| 2. Please include a complete copy of PLOS’ questionnaire on inclusivity in global research in your revised manuscript. Our policy for research in this area aims to improve transparency in the reporting of research performed outside of researchers’ own country or community. The policy applies to researchers who have travelled to a different country to conduct research, research with Indigenous populations or their lands, and research on cultural artefacts. The questionnaire can also be requested at the journal’s discretion for any other submissions, even if these conditions are not met.  Please find more information on the policy and a link to download a blank copy of the questionnaire here: <https://journals.plos.org/plosone/s/best-practices-in-research-reporting>. Please upload a completed version of your questionnaire as Supporting Information when you resubmit your manuscript. | A copy of PLOS’ questionnaire on inclusivity in global research has been completed and submitted with our revised manuscript. |
| 3. In your Data Availability statement, you have not specified where the minimal data set underlying the results described in your manuscript can be found. PLOS defines a study's minimal data set as the underlying data used to reach the conclusions drawn in the manuscript and any additional data required to replicate the reported study findings in their entirety. All PLOS journals require that the minimal data set be made fully available. For more information about our data policy, please see <http://journals.plos.org/plosone/s/data-availability>.  Upon re-submitting your revised manuscript, please upload your study’s minimal underlying data set as either Supporting Information files or to a stable, public repository and include the relevant URLs, DOIs, or accession numbers within your revised cover letter. For a list of acceptable repositories, please see <http://journals.plos.org/plosone/s/data-availability#loc-recommended-repositories>. Any potentially identifying patient information must be fully anonymized.  Important: If there are ethical or legal restrictions to sharing your data publicly, please explain these restrictions in detail. Please see our guidelines for more information on what we consider unacceptable restrictions to publicly sharing data: <http://journals.plos.org/plosone/s/data-availability#loc-unacceptable-data-access-restrictions>. Note that it is not acceptable for the authors to be the sole named individuals responsible for ensuring data access.  We will update your Data Availability statement to reflect the information you provide in your cover letter. | The minimal data set underlying the results reported in this paper are included as supporting information files. |
