# Supplementary material for: Experiences of redeployed healthcare workers in the fight against COVID-19 in China: A qualitative study
Source: PLoS One. 2022 Aug 25;17(8):e0273429. doi: 10.1371/journal.pone.0273429 (PMC9409527; doi:10.1371/journal.pone.0273429)
Supplement: Attachment — Submitted filename: Response to reviewers 06.08.2022.docx [file pone.0273429.s004.docx]

**PONE-D-21-40460R1**

**Experiences of redeployed healthcare workers in the fight against COVID-19 in China:**

**a qualitative study**

Response to editors/reviewers

| **Reviewers’ comments** | **Authors’ response and changes made** |
| --- | --- |
| Journal Requirements: | |
| We recommend that you either get your manuscript reviewed by someone who is fluent in English or, if you would like professional help, you can use any reputable English language editing service. | Thank you so much for your suggestion. Our manuscript has been critically reviewed and thoroughly edited by someone who is fluent in English. All the changes made have been highlighted using track changes. |
| Please review your reference list to ensure that it is complete and correct. If you have cited papers that have been retracted, please include the rationale for doing so in the manuscript text, or remove these references and replace them with relevant current references. Any changes to the reference list should be mentioned in the rebuttal letter that accompanies your revised manuscript. If you need to cite a retracted article, indicate the article’s retracted status in the References list and also include a citation and full reference for the retraction notice. | We have carefully reviewed our reference list to ensure that it is complete and correct. Two duplicates were identified and removed. In addition, we would like to inform you the changes to the reference list below.  (1) In our previous manuscript, we cited one paper that has been retracted:  30. Panagioti M, Geraghty K, Johnson J, Zhou A, Panagopoulou E, Chew-Graham C, et al. Association between physician burnout and patient safety, professionalism, and patient satisfaction: a systematic review and meta-analysis. JAMA internal medicine. 2018;178(10):1317-31.  Now we removed this reference and replaced it with another relevant current reference below:  30. Salyers MP, Bonfils KA, Luther L, Firmin RL, White DA, Adams EL, et al. The relationship between professional burnout and quality and safety in healthcare: A meta-analysis. J Gen Intern Med. 2017;32(4):475-82.  (2) The previous reference 20 - a government press conference is not available to track the relevant information:  20. China NHCotPsRo. Press conference of the joint prevention and control mechanism of the State Council on Jan 26, 2020.  Therefore, we have removed this reference and replaced it with another relevant current reference providing high quality evidence.  20. Jiang L, Broome ME, Ning C. The performance and professionalism of nurses in the fight against the new outbreak of COVID-19 epidemic is laudable. Int J Nurs Stud. 2020;107:103578.  (3) The following sentence in the discussion section did not have a reference to support in our previous manuscript, therefore, we added a relevant current reference below:  Research during previous infectious disease outbreaks has shown that ample supplies of PPE significantly facilitated effective clinical care, while insufficient or rapidly depleting PPE contributed to healthcare workers’ anxiety [70].  70. Cox M, Sharma D, Phillips G, Mitchell R, Herron LM, Brolan CE, et al. Lessons from the frontline: Documenting the pandemic emergency care experience from the Pacific region - Infrastructure and equipment. Lancet Reg Health West Pac. 2022:100516. |
| Reviewers' comments: | |
| Reviewer #2: All comments have been addressed  I have read thoroughly the revised version of paper. Now this version of the paper after revision done is OK and provides interesting results for readers of PLOS ONE. | Thank you so much for your positive comments.  We are very pleased to hear from you that we have addressed all your previous comments and this manuscript provides interesting results for readers of PLOS ONE. |
| Reviewer #3: All comments have been addressed  The authors have successfully addressed the comments. The manuscript can be considered for publication. | Thank you so much for your positive comments.  We are very pleased to hear from you that we have successfully addressed your previous comments, and you recommend it to be published in PLOS ONE. |
